# Carotid artery intima-media thickness in women with polycystic ovary syndrome: A cross-sectional study

**DOI:** 10.18502/ijrm.v22i9.17480

**Published:** 2024-11-14

**Authors:** Nazanin Farshchian, Parisa Bahrami Kamangar, Mohammad Reza Ghasempour

**Affiliations:** Radiology Department, Clinical Research Development Center, Imam Reza Hospital, Kermanshah University of Medical Sciences, Kermanshah, Iran.

**Keywords:** Polycystic ovary disease, Atherosclerosis, Intima media thickness, Carotid artery, Cardiovascular disease.

## Abstract

**Background:**

Obesity in polycystic ovary syndrome (PCOS) may increase the risk of cardiovascular disease. Carotid intima-media thickness (CIMT) is an acceptable marker in assessing the risk of heart disease.

**Objective:**

This study aimed to evaluate the CIMT in PCOS women compared to non-PCOS women.

**Materials and Methods:**

This cross-sectional study was conducted on 48 women who referred to Imam Reza hospital, Kermanshah, Iran from July 2020–2021. Women were divided into 2 groups of PCOS and non-PCOS women (n = 24/each). The intima-media thickness of participants' carotid artery was measured on both sides in 3 areas, and its mean was recorded.

**Results:**

The mean thickness of the carotid artery intima-media in the case group was within the normal range; but it was significantly higher than the control group (0.71 ± 0.17 vs. 0.57 ± 0.09 mm, p = 0.019).

**Conclusion:**

Considering the higher thickness of CIMT in women with PCOS, it can be concluded that PCOS increases the risk of heart diseases in women.

## 1. Introduction

Polycystic ovarian disease (PCOD) is one of the most common endocrine disorders in women of reproductive age with symptoms of enlarged and fibrotic ovaries, menstrual irregularities, obesity, hirsutism, lack of ovulation, and infertility (1–5). In the ultrasound examination, with the best sensitivity and specificity, the ovarian volume of 10.5 ml along with 12 or more 2–9 mm follicles has been introduced to the diagnosis of PCOD (6).

In addition, uterine artery and ovarian stromal blood flow parameters in women with polycystic ovary syndrome (PCOS) are different from non-PCOS women (7). If these sonographic symptoms are accompanied by symptoms of hyperandrogenism, it is called PCOS (8). According to the diagnostic criteria, the PCOS prevalence in the world varies between 2.2 and 20% (2, 3, 9). In Iran, according to the Rotterdam criteria, the PCOD prevalence has been reported up to 19.5% (10).

The exact causes of PCOS remain unclear. However, commonly cited factors include lifestyle, genetic predisposition, insulin-related issues, obesity, environmental and chemical pollution, high blood pressure, hyperlipidemia, and increased abdominal fat (11, 12). All these cases are somehow associated with the risk of developing atherosclerosis (13). Atherosclerosis and other arterial diseases can result in serious health conditions such as heart failure, stroke, kidney issues, organ ischemia, and even death, particularly prevalent in developed nations (14). Ultrasound is a simple and non-invasive imaging technique for assessing carotid artery intima-media thickness (CIMT). The increase in CIMT is one of the most important endothelial structural changes occurring early in atherosclerosis. Ultrasound can detect and monitor it, serving as an initial indicator for atherosclerosis diagnosis (15). Given the association of PCOS with hypertension, hyperlipidemia, increased abdominal fat, and obesity, the risk of atherosclerosis is heightened. There are conflicting research findings in this field (16, 17).

Therefore, this study aimed to compare CIMT in PCOS women with non-PCOS women.

## 2. Materials and Methods 

In this cross-sectional study, 48 women referred to Imam Reza hospital, Kermanshah, Iran from July 2020–2021 were enrolled. Participants were divided into 2 groups of case and control (n = 24).

The case group consisted of women who were diagnosed with PCOS by a gynecologist, based on the Rotterdam criteria (2 of 3 of the following criteria showing irregular menstrual cycles), obesity (body mass index [BMI] ≥ 30), and hirsutism, hormonal tests (indicating signs of hyperandrogenism), and sonography (revealing an ovarian volume of 10.5 ml and 12 or more 2–9 mm follicles) (18).

Menstrual irregularity was considered as an interval between periods of < 21 or > 35 days. The control group consisted of non-PCOS women aged between 25 and 40 yr, without clinical or sonographic signs of polycystic ovary, is included.

The exclusion criteria were a past medical history of diabetes, severe kidney conditions (chronic renal failure), heart diseases, chronic hypertension, ovarian and adrenal tumors, congenital adrenal hyperplasia, smoking, and lack of consent to participate in the study.

The CIMT of all women was measured in at least 3 areas of the common carotid artery on both sides by an expert radiologist with SAMSUNG Medison Co. WS80A (2015) Korean ultrasound machine and 7–12 MHz linear probe and its average was recorded. Sonography results, including CIMT, along with BMI, height, weight, and age of the participants, were recorded and compared between the 2 groups.

### Sample size

The sample size for each group was calculated to be 24 in each group, considering a 95% confidence level and the power of 90%, referencing a prior study (19) and the formula below:

$N={\left(Z_{1-\alpha /2}+Z_{1-\beta }\right)}^{2}{\left(\sigma_{1}^{2}+\sigma_{2}^{2}\right)/\left(\mu_{1}-\mu_{2}\right)}^{2}$

${\left(1.96+1.28\right)}^{2}\left(7.3^{2}+7.6^{2}\right)/{\left(56.8-49.8\right)}^{2}=23.8$

### Ethical Considerations

This study was approved by the Ethics Committee of Kermanshah University of Medical Sciences, Kermanshah, Iran (Code: IR.KUMS.REC.1398.843). After completing the informed written consent form, the women entered the study.

### Statistical Analysis

For all statistical analysis, SPSS, software (version 16.0, SPSS Inc., Chicago, Illinois, USA) was used. Quantitative data analysis was done using mean and standard deviation and qualitative data analysis was done using mean and standard deviation. To check the normality of quantitative variables, the Kolmogorov–Smirnov test was used. Based on the results of the Kolmogorov–Smirnov test, Pearson's correlation coefficient was used to calculate the relationship between intima-media thickness and height, weight, age, and BMI. In addition, the student's *t* test was used to compare quantitative data in 2 groups. P < 0.05 were considered significant.

## 3. Results

From a total of 73 women, 48 women were enrolled in the present study. Participants were divided into 2 groups: the case group (PCOS women) and the control group (non-PCOS women). 10 participants from the case group and 15 from the control group were excluded due to lack of consent to participate in the study and mentioned exclusion criteria. Ultimately, data of 48 women (24 in each group) were analyzed.

The women in the case and control groups had a similar mean age. The mean BMI of the women in the case and control groups were 29.15 ± 1.99 and 29.93 ± 1.19 kg/m^2^, respectively (p = 0.023). However, CIMT was significantly higher in the case group compared to the controls (Table I). No significant relationship was observed between CIMT with age (R = 0.274, p = 0.161), weight (R = 0.146, p = 0.213), height (R = 0.716, p = 0.054), and BMI (R = 0.257, p = 0.077) (Table II).

**Table 1:** Comparison of the age, BMI, and CIMT between groups

**Variables**	**Min**	**Max**	**Groups**		**P-value**
			**Control )n = 24)**	**Case )n = 24)**	
**Age (yr)**	25	40	33.17± 3.91	33.12 ± 3.81	0.726
**BMI (kg/m^2^)**	24.49	33.18	29.93 ± 1.19	29.15 ± 1.99	0.023
**CIMT (mm)**	0.4	1.1	0.57± 0.09	0.71 ± 0.17	0.019
Data presented as Mean ± SD, Student’s t test. BMI: Body mass index, CIMT: Carotid artery intima-media thickness					

**Table 2:** Correlation of CIMT with BMI, age, weight, and height

**CIMT**	**BMI**	**Age**	**Weight**	**Height**
**< 30**	Correlation coefficient test	-0.012	0.038	-0.041
	P-value	0.954	0.852	0.843
**≥ 30**	Correlation coefficient test	0.299	0.292	0.002
	P-value	0.177	0.187	0.993
CIMT: Carotid artery intima-media thickness, BMI: Body mass index, Pearson's correlation coefficient test				

## 4. Discussion

According to the findings of the present cross-sectional study, CIMT significantly increased in PCOD patients. This increase was independent of BMI, age, weight, and height and our findings suggest that the disorder itself has a causative role in CIMT increase. CIMT measurement using B-mode ultrasound is a valuable marker of subclinical atherosclerosis and represents the risk of cardiovascular disease.

According to some previous studies, the mean CIMT in PCOS women is markedly higher than control groups (19–22). In other studies, there is no obvious evidence of increased CIMT in PCOS women (16, 17). Obesity and central body fat distribution regardless of BMI have been linked to increase CIMT, and both are common findings in PCOS women (23, 24).

In addition, increased visceral fat enhances the secretion of free fatty acids and inflammatory cytokines (25). These substances have an important effect on metabolism and the cardiovascular system. Visceral fat is also associated with an increased risk of cardiovascular diseases and dyslipidemia (26).

However, in our study, no obvious differences are seen between BMI of case and control groups, but PCOS patients have central body fat distribution regardless of BMI. Androgen levels have a positive association with CIMT, and PCOS patients have high levels of androgen, so it is suggested that CIMT increase in PCOS patients is related to hyperandrogenism (27).

Although there was no history of smoking in any of our case and control groups, and chronic hypertension was one of the exclusion criteria, smoking and hypertension are also related to increased CIMT (28, 29).

Finally, our results are in accordance with previous studies in which PCOS women had increased CIMT. In addition, variations in results of some of the previous studies could be due to differences in PCOS duration, age, and genetics of patients or sample size. The strength of this study is comparable age, height, weight, and BMI in case and control groups. Limitation of the current study was the small sample size. Further studies with a larger sample size are suggested.

Based on the results of our study, the CIMT was significantly higher in the PCOD group. Considering that the increase in the thickness of the carotid artery intima-media is a marker for atherosclerosis and the prediction of cardiovascular diseases, it is suggested that PCOD women be examined for cardiovascular diseases.

##  Data Availability

Data supporting the findings of this study are available upon reasonable request from the corresponding author.

##  Author Contributions

N. Farshchian designed the study and conducted the research. M.R. Ghasempour and P. Bahrami Kamangar drafted the manuscript and reviewed the article. All authors approved the final manuscript and take responsibility for the integrity of the data.

##  Conflict of Interest

The authors declare that there is no conflict of interest.
